# Elevated serum omentin levels correlate with tumor aggressiveness and disease progression in breast cancer patients

**DOI:** 10.3389/fonc.2026.1774692

**Published:** 2026-03-30

**Authors:** Himanshu Raj, Priyamvada Gupta, Vibhav Gautam, Sanjeev Kumar Gupta

**Affiliations:** 1 Department of General Surgery, Institute of Medical Sciences, Banaras Hindu University, Varanasi, India; 2 Centre of Experimental Medicine and Surgery, Institute of Medical Sciences, Banaras Hindu University, Varanasi, India

**Keywords:** adipokine, biomarker, breast cancer, ELISA, HER2, inflammation, obesity, omentin

## Abstract

Breast cancer remains the most frequently diagnosed malignancy among women globally and a leading cause of cancer related mortality. Despite significant progress in diagnosis and treatment, the disease’s heterogeneity continues to challenge effective management. Recent studies suggest that metabolic dysfunction and obesity related factors contribute significantly to breast cancer risk and progression. Among various adipokines secreted by adipose tissue, omentin has emerged as a novel molecule of interest due to its insulin sensitizing and anti-inflammatory properties. This study explores the clinical relevance of serum omentin levels in breast cancer patients by comparing them with healthy controls and examining their correlation with different tumor characteristics. Serum omentin levels were significantly higher in breast cancer patients compared to those with benign breast conditions, which in turn were higher than in healthy controls. The study demonstrated a meaningful association between serum omentin levels and key clinicopathological parameters such as age, BMI, tumor size, lymph node status, tumor grade, and hormone receptor expression, reflecting its possible role in indicating disease severity and tumor biology. The findings may enhance our understanding of the metabolic tumor interface and support the potential of omentin as a diagnostic or prognostic biomarker.

## Introduction

Breast cancer is the most prevalent cancer among women worldwide, with over two million new cases annually. Despite advancements in surgical techniques, chemotherapy, hormonal therapy, and targeted interventions, breast cancer remains a leading cause of cancer related deaths due to its heterogeneous nature (1). Variability in tumor histopathology, genetic mutations, and receptor status (e.g., ER, PR, HER2) complicates prognosis and therapeutic responses (2). While molecular subclassification into luminal A, luminal B, HER2 enriched, and triple negative types aids in clinical decision making, it does not fully capture the diverse biological behaviours of breast cancer (3). Granular cell tumor (GCT) is a rare neoplasm of the soft tissues that is commonly found in the tongue, but can rarely affect rectus abdominis muscle (4) and breast in females (5). The clinical and radiological resemblance of breast granular cell tumors to malignant breast carcinoma leads to potential misdiagnosis.

Emerging research has highlighted the importance of metabolic dysregulation and obesity in breast cancer pathogenesis (6). Adipose tissue, once regarded solely as an energy reservoir, is now recognized as a dynamic endocrine organ that secretes adipokines, bioactive molecules involved in inflammation, insulin resistance, and tumorigenesis (7). Among these, omentin 1 (also known as intelectin-1) has attracted attention due to its anti-inflammatory and insulin sensitizing effects. Predominantly secreted by visceral fat, omentin may influence cancer biology by modulating systemic metabolic and inflammatory pathways (8).

Although preliminary studies suggest a potential tumor suppressive role for omentin, its function in breast cancer remains poorly understood. Investigating serum omentin levels in breast cancer patients may provide new insights into the metabolic underpinnings of disease progression and could help identify novel diagnostic and prognostic biomarkers. This study aims to compare serum omentin concentrations between breast cancer patients and healthy controls and to assess correlations with key clinical and pathological features, thereby exploring omentin’s potential clinical utility in breast cancer management.

## Material and methods

### Study design and setting

This institution-based, prospective observational study was conducted over a period of 24 months, from April 2023 to March 2025, in the Department of General Surgery and the Centre of Experimental Medicine & Surgery (CEMS), Institute of Medical Sciences, Banaras Hindu University (BHU), Varanasi. The study was designed to evaluate the clinical significance of serum omentin levels in breast cancer patients in comparison with patients with benign breast disease and healthy controls.

### Study population

Female participants aged 18 years or older were included in the study. The population was categorized into three groups: forty histologically confirmed breast cancer patients, twenty patients with benign breast disease, and twenty age-matched healthy female volunteers with no history of malignancy. Ethical approval was obtained from the Institute Ethics Committee of IMS-BHU, and written informed consent was secured from all participants prior to inclusion in the study. Patients were excluded if they had previously undergone chemotherapy, radiotherapy, or any surgical intervention for breast malignancy, or if they presented with metastatic disease at the time of evaluation.

### Clinical and diagnostic evaluation

All participants underwent a detailed clinical evaluation, including a thorough history and physical examination. Imaging modalities such as ultrasonography and/or mammography were employed. The diagnosis was confirmed through fine needle aspiration cytology (FNAC) and/or core needle biopsy, forming a triple assessment approach.

### Tumor grading

Tumor grading was performed using the Bloom-Richardson grading system (9). This histopathological method assesses three morphological parameters: tubule formation, nuclear pleomorphism, and mitotic count, each scored from 1 to 3. The sum of these scores yields a total between 3 and 9, which determines tumor grade as follows: Grade I (well differentiated, score 3–5), Grade II (moderately differentiated, score 6–7), and Grade III (poorly differentiated, score 8–9). This grading system provided valuable prognostic information.

### Immunohistochemistry

Immunohistochemical analysis was carried out on biopsy tissue samples to evaluate the expression of estrogen receptor (ER), progesterone receptor (PR), and HER2/neu. Based on these receptor profiles, tumors were further classified into molecular subtypes, supporting both prognostic evaluation and treatment planning.

### Blood collection and serum preparation

Fasting venous blood samples were collected from all participants. The blood samples were centrifuged at 3000 rpm for 10 minutes to separate the serum. The obtained serum was divided into aliquots and stored at –80 °C for subsequent biochemical analysis.

### Estimation of serum omentin (ITLN1) levels

Serum omentin levels were quantified using a Human ITLN1/Omentin ELISA Kit (FineTest), employing a sandwich enzyme-linked immunosorbent assay method according to the manufacturer’s instructions. In brief, 100 µL of serum was added to wells of a 96-well plate pre-coated with anti-ITLN1 antibody and incubated at 37 °C for 90 minutes. After initial incubation, the wells were washed, followed by the addition of biotin-labelled detection antibody and incubation at 37 °C for 60 minutes. The plate was then washed and incubated with HRP-conjugated Streptavidin for 30 minutes. After multiple washings, TMB substrate solution was added and incubated for 10–20 minutes at 37 °C. The reaction was terminated with stop solution, and absorbance was measured at 450 nm using a microplate reader. Serum omentin concentrations were calculated based on a standard curve generated with known concentrations (**Supplementary Figure 1**).

### Statistical analysis

All statistical analyses were performed using GraphPad Prism version 8.0.2. Serum omentin levels were expressed as mean ± SEM. Data normality and variance homogeneity were assessed before applying tests. One-way ANOVA followed by Tukey’s *post hoc* test was used to compare omentin levels among cancer, benign, and healthy groups. Within the cancer group, independent *t*-tests and one-way ANOVA were used for binary and multi-category variables, respectively. Pearson or Spearman correlation was applied for continuous variables (e.g., age, BMI). Paired *t*-tests assessed pre- and post-treatment changes. The following thresholds were used to denote levels of statistical significance: *p* ≤ 0.033 (significant, *), *p* ≤ 0.002 (highly significant, **), and *p* ≤ 0.001 (very highly significant, ***).

## Results

### Demographic distribution of study participants

The study comprised a total of 80 female participants, including 40 histologically confirmed breast cancer patients, 20 patients with benign breast disease, and 20 healthy controls. Age-wise distribution revealed that the majority of breast cancer patients (50%) were aged above 45 years, whereas in the benign group, 35% were >45 years, and among healthy controls, this proportion was 45%. Notably, younger participants (<30 years) were more frequently represented in the benign group (25%) compared to cancer and healthy groups (10% each). The 30–45 years age group showed relatively even distribution across all three categories.

### Clinical characteristics of breast cancer patients

Among the 40 breast cancer patients, 55% had tumors measuring ≥5 cm in size, while 45% had tumors <5 cm. A significant proportion (80%) were node-positive (N1), whereas only 20% were node-negative (N0). In terms of clinical staging, 70% of patients had locally advanced breast cancer (Stage IIB–III), while 30% were diagnosed at early stages (Stage I–IIA).

### Histopathological and molecular parameters

Histological grading showed that 50% of patients had poorly differentiated (Grade 3) tumors, 38% had moderately differentiated (Grade 2), and only 13% had well-differentiated (Grade 1) tumors. Lymphovascular invasion (LVI) was observed in 33% of the patients. Estrogen receptor (ER) positivity was found in 45% of cases, progesterone receptor (PR) positivity in 40%, and HER2 overexpression in 35% of patients. Based on molecular classification, Luminal B was the most common subtype (30%), followed by Luminal A (25%), Triple-Negative Breast Cancer (TNBC) (25%), and HER2-enriched (20%).

### Association of omentin levels with age and BMI

**Figure 1:**
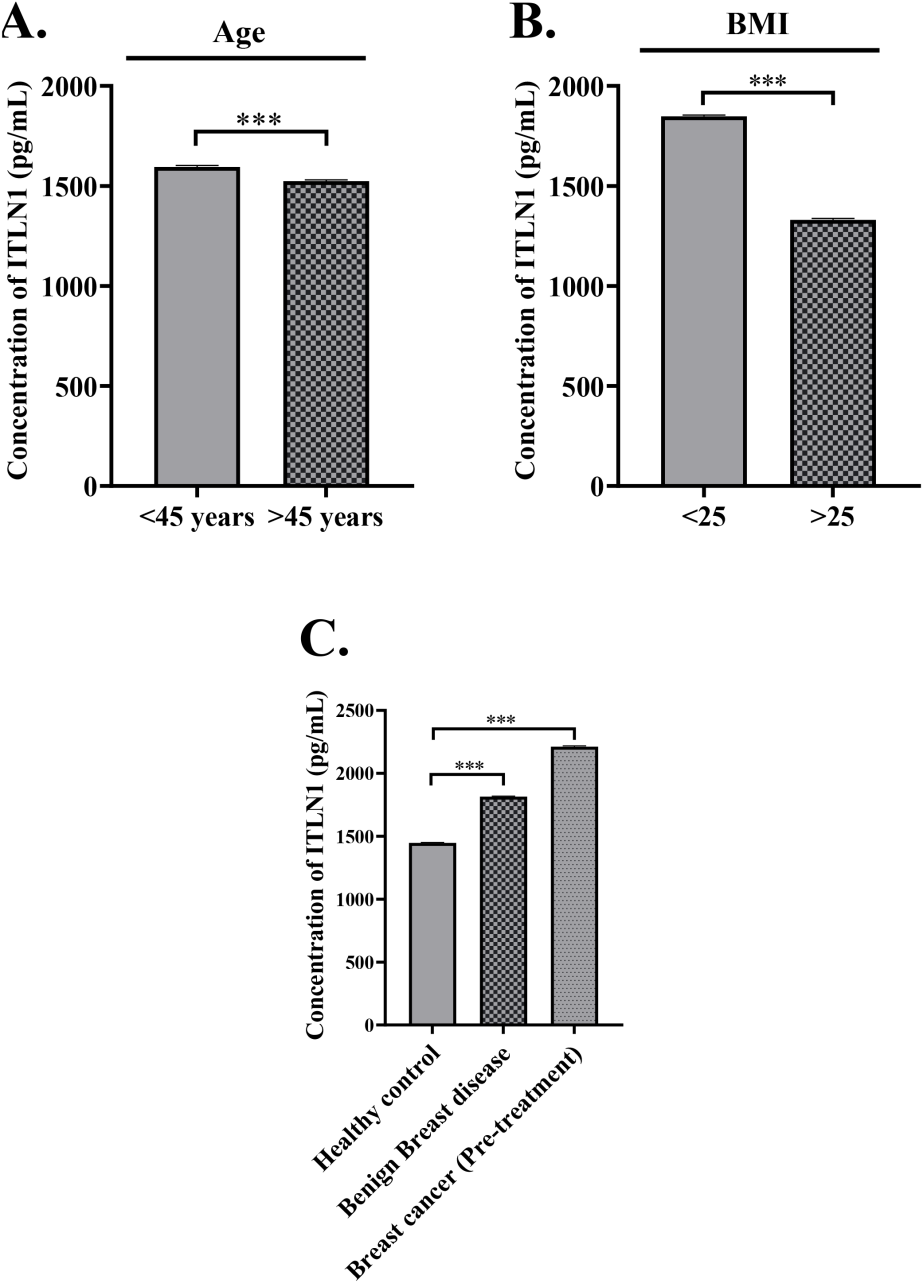
**(A)** Patients aged >45 years exhibited lower mean Omentin concentrations compared to those aged <45 years indicating a potential inverse relationship between age and circulating Omentin levels in the overall study population. **(B)** Patients with BMI >25 exhibited lower mean Omentin concentrations compared to those with BMI <25, suggesting an inverse correlation between BMI and circulating Omentin levels. **(C)** Cancer patients exhibited the highest mean Omentin levels followed by benign cases, while healthy controls had the lowest levels showing potential association between elevated Omentin levels and the presence of malignancy. The data is representative of three independent experiments and expressed as means ± SEM. The statistical significance (***p ≤0.001, **p ≤0.002, *p ≤0.033) was calculated using one-way ANOVA and Unpaired *t-test.*.

Analysis revealed a significant inverse relationship between age and circulating omentin levels. Patients under 45 years exhibited higher mean omentin levels (1595.33 ± 6.63 pg/mL) than those aged >45 years (1524.59 ± 6.00 pg/mL; p < 0.001) (**Figure 1A**). Similarly, individuals with BMI <25 kg/m² had significantly higher serum omentin levels (1848.10 ± 6.0 pg/mL) compared to those with BMI ≥25 kg/m² (1330.63 ± 6.63 pg/mL; p < 0.001) (**Figure 1B**), indicating a strong inverse association between BMI and omentin levels.

### Comparison of omentin levels across study groups

Serum omentin concentrations varied significantly across the three study groups. Breast cancer patients demonstrated the highest mean omentin levels (2212.60 ± 5.56 pg/mL), followed by patients with benign disease (1814.52 ± 4.47 pg/mL), while healthy controls had the lowest levels (1446.71 ± 4.47 pg/mL) (**Figure 1C**). These differences were statistically significant (p < 0.001), indicating a strong association between elevated omentin levels and the presence of breast pathology.

### Correlation of omentin levels with tumor size, nodal status, and stage

**Figure 2:**
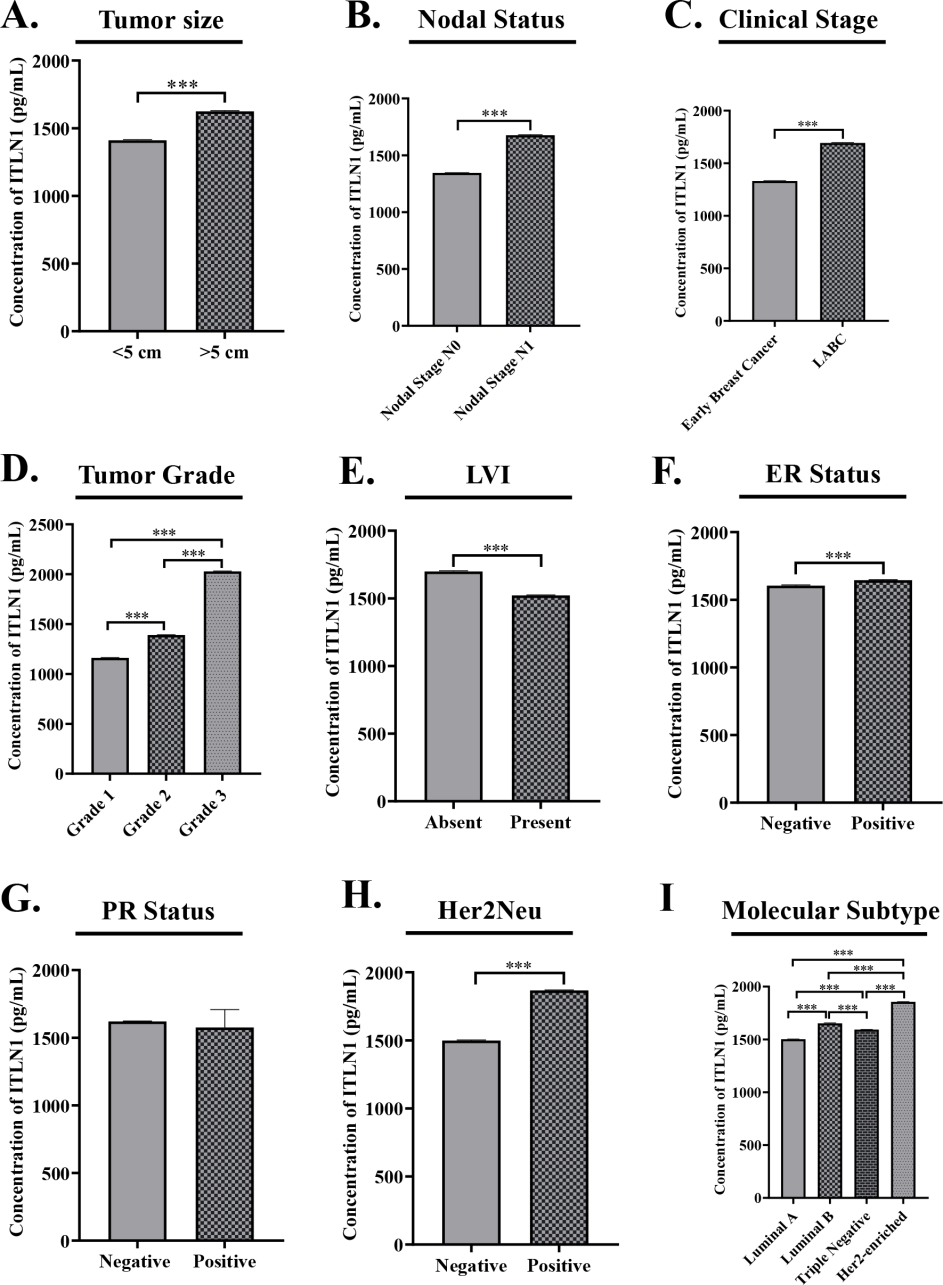
**(A)** Patients with larger tumors (≥5 cm) demonstrated higher mean serum Omentin levels compared to patients with smaller tumors (<5 cm) suggesting a positive association between tumor size and circulating Omentin concentrations. **(B)** Patients with lymph node involvement (N1) exhibited higher mean serum Omentin levels compared to those without palpable lymph nodes (N0). **(C)** Patients with early-stage disease (Stage I–IIA) exhibited lower mean serum Omentin levels compared to those with locally advanced cancer (Stage IIB–III). **(D)** Patients with high-grade tumor (Grade 3) exhibited the highest mean Omentin concentration which was significantly greater than levels observed in Grade 2 and Grade 1 tumor. **(E)** Lower mean serum Omentin levels was observed in patients with LVI than those without LVI. **(F)** Estrogen receptor (ER) negative patients showed lower serum Omentin level than that of ER positive patients. **(G)** PR-positive individuals exhibit slightly lower serum Omentin level as compared to PR-negative patients. **(H)** HER2-positive patients demonstrated markedly elevated omentin-1 levels in comparison to HER2-negative patients. **(I)** Patients with the HER2-enriched subtype exhibited the highest omentin-1 levels followed by Luminal B, Triple negative and Luminal A. The data is representative of three independent experiments and expressed as means ± SEM. The statistical significance (***p ≤0.001, **p ≤0.002, *p ≤0.033) was calculated using one-way ANOVA and Unpaired *t-test.*.

Within the breast cancer group, patients with tumors ≥5 cm showed significantly higher serum omentin levels (1625.14 ± 5.09 pg/mL) than those with tumors <5 cm (1410.73 ± 4.00 pg/mL; p = 0.001) (**Figure 2A**). Node-positive patients had elevated omentin levels (1676.79 ± 5.38 pg/mL) compared to node-negative individuals (1344.90 ± 2.82 pg/mL; p < 0.001) (**Figure 2B**). Similarly, locally advanced breast cancer patients had significantly higher omentin levels (1692.54 ± 5.19 pg/mL) than those with early-stage disease (1330.08 ± 3.61 pg/mL; p < 0.001) (**Figure 2C**), suggesting a link between omentin expression and disease progression.

### Omentin levels in relation to tumor grade and lymphovascular invasion

A clear association was found between tumor grade and serum omentin levels. Patients with Grade 3 tumors had the highest omentin levels (2028.21 ± 3.4 pg/mL), followed by Grade 2 (1389.91 ± 3.1 pg/mL) and Grade 1 tumors (1161.76 ± 2.0 pg/mL) (**Figure 2D**). This difference was statistically significant (p < 0.001). Interestingly, patients with LVI had lower omentin levels (1521.96 ± 3.31 pg/mL) compared to those without LVI (1699.14 ± 4.69 pg/mL) (**Figure 2E**), also statistically significant (p < 0.001).

### Omentin levels based on ER, PR, and HER2 status

Serum omentin levels were significantly higher in ER-positive patients (1644.89 ± 3.74 pg/mL) compared to ER-negative patients (1604.45 ± 5.09 pg/mL; p < 0.01) (**Figure 2F**). No significant difference was noted with PR status (p = 0.826) (**Figure 2G**). However, HER2-positive patients had significantly elevated omentin levels (1866.66 ± 3.6 pg/mL) compared to HER2-negative patients (1498.36 ± 5.0 pg/mL; p < 0.001) (**Figure 2H**), suggesting an association between omentin and HER2 overexpression.

### Serum omentin across molecular subtypes of breast cancer

When analyzed according to molecular subtypes, the HER2-enriched group showed the highest serum omentin levels (1855.64 ± 2.44 pg/mL), followed by Luminal B (1652.4 ± 3.60 pg/mL), TNBC (1592.42 ± 3.0 pg/mL), and Luminal A (1501.36 ± 2.82 pg/mL) (**Figure 2I**). These differences were statistically significant (p < 0.001), suggesting subtype-specific patterns of omentin expression.

### Effect of treatment on serum omentin levels

**Figure 3:**
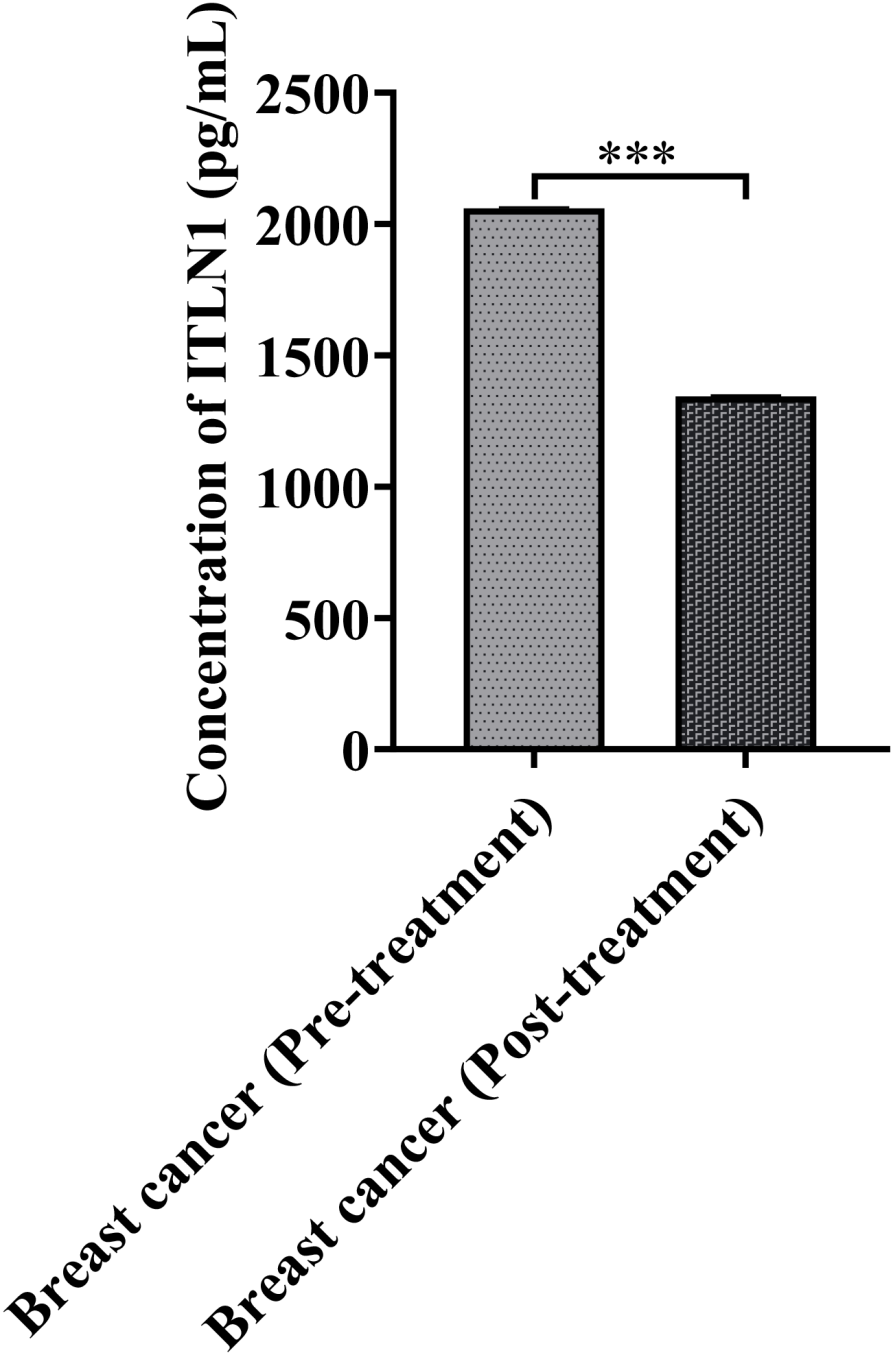
The mean Omentin concentration in breast cancer patients decreased post-treatment as compared to pre-treatment. The data is representative of three independent experiments and expressed as means ± SEM. The statistical significance (***p ≤0.001, **p ≤0.002, *p ≤0.033) was calculated using Unpaired *t-test.*.

A subset of 24 breast cancer patients was assessed for pre- and post-treatment serum omentin levels. A significant decline in omentin levels was observed following treatment. Pre-treatment mean levels were 2059.68 ± 4.0 pg/mL, which dropped to 1344.40 ± 4.0 pg/mL post-treatment (p < 0.001) (**Figure 3**). This indicates that therapeutic intervention may impact circulating omentin levels, potentially serving as a biomarker for treatment response.

## Discussion

This prospective observational study included 80 female participants, comprising 40 breast cancer patients, 20 individuals with benign breast disease, and 20 healthy controls. The age distribution revealed a higher proportion of breast cancer patients above 45 years, consistent with the known increased risk of breast cancer with advancing age. Conversely, benign breast disease was more common among younger women, reflecting the prevalence of hormone-driven conditions like fibroadenomas in this group. A significant inverse association was found between serum omentin levels and both age and body mass index (BMI). These findings are in line with prior research reporting that younger and leaner individuals tend to have higher circulating omentin. Though studies have shown conflicting results on BMI’s role in breast cancer development, it remains a clinically important factor, particularly in its influence on adipokines like omentin.

Serum omentin levels were significantly elevated in breast cancer patients compared to benign and healthy groups, suggesting a potential role of omentin in tumor-associated metabolic stress or inflammation. The progressive rise in omentin levels from healthy individuals to benign and malignant groups implies its involvement in tumor biology. While some researchers suggest this elevation might support tumor cell metabolism and growth (8, 10), others have found decreased omentin levels in breast cancer, suggesting a context-dependent tumor-suppressive function (11–13). Higher omentin concentrations were also observed in patients with larger tumors (≥5 cm), lymph node metastasis, and more advanced tumor stages. These associations point toward a possible relationship between omentin expression and tumor burden or metastatic potential, possibly mediated by inflammatory or metabolic signaling pathways. Similarly, patients with high-grade (Grade 3) tumors showed the highest omentin levels, supporting its association with aggressive histological features and possibly increased cellular turnover or metabolic demand.

Serum omentin levels also varied with molecular characteristics. HER2-positive patients exhibited significantly higher levels than HER2-negative individuals, possibly due to the pro-inflammatory environment associated with HER2 overexpression. Estrogen receptor (ER) positivity was also linked with higher omentin levels, suggesting a potential association with favorable hormone receptor status. The high levels of serum omentin level in ER-positive patients is not ideally reported, however, a study reports similar findings in postmenopausal breast cancer patients (12). The estrogen receptors are present in adipose tissue and therefore it may be involved in the direct or indirect regulation of adipokines, including Omentin. Based on the evidences, it is thus assumed that estrogen may act as a key player in elevating serum omentin level and thus emphasize the necessity for further investigation. These patterns suggest that omentin expression may be influenced by tumor subtype and underlying molecular behavior.

A key observation of this study was the significant decline in serum omentin levels following treatment in breast cancer patients. This decrease may reflect a reduction in tumor burden, systemic inflammation, or metabolic dysregulation following therapy. The dynamic nature of this change supports the hypothesis that omentin could serve as a biomarker to monitor treatment response. However, some studies have reported lower baseline omentin levels in cancer patients, suggesting it may have tumor-suppressive effects in certain settings. These contradictory findings may reflect differences in study populations or the dual biological role of omentin depending on tumor microenvironment and metabolic status.

In summary, this study highlights that elevated serum omentin is significantly associated with advanced disease features and declines after treatment, suggesting its potential as a diagnostic, prognostic, and treatment-responsive biomarker in breast cancer. Further large-scale and mechanistic studies are necessary to better understand its clinical utility.

## Data Availability

The original contributions presented in the study are included in the article/**Supplementary Material**. Further inquiries can be directed to the corresponding author.
