# Supplementary material for: Elevated serum omentin levels correlate with tumor aggressiveness and disease progression in breast cancer patients
Source: Front Oncol. 2026 Mar 30;16:1774692. doi: 10.3389/fonc.2026.1774692 (PMC13070900; doi:10.3389/fonc.2026.1774692)
Supplement: Supplementary file 1 [file DataSheet1.docx]

**Supplementary Information**

**Title:** Elevated Serum Omentin Levels Correlate with Tumor Aggressiveness and Disease Progression in Breast Cancer Patients

**Authors**: Himanshu Raj^1^, Priyamvada Gupta^2^, Vibhav Gautam^2^, Sanjeev Kumar Gupta*^1^

**Affiliations**

*^1^Department of General Surgery, Institute of Medical Sciences, Banaras Hindu University, Varanasi-221005, India*

*^2^Centre of Experimental Medicine and Surgery, Institute of Medical Sciences, Banaras Hindu University, Varanasi-221005, India*

*** Corresponding author**

Prof. Sanjeev Kumar Gupta

**Address**

Department of General Surgery

Institute of Medical Sciences

Banaras Hindu University

Varanasi 221005, India

Telephone: +919415203087

Email ID:  [drsanjeevkgupta@gmail.com](mailto:drsanjeevkgupta@gmail.com)

**Number of Figure: 01**

**Figure S1.** Calibration curve of Intelectin-1 for estimating serum omentin
